# β-Arrestin 2 suppresses the activation of YAP by promoting LATS kinase activity

**DOI:** 10.1016/j.gendis.2022.04.017

**Published:** 2022-05-17

**Authors:** Minsuh Kim, Ji Min Kim, Eun Jeong Cho, Chang Ohk Sung, Joon Kim, Se Jin Jang

**Affiliations:** a Asan Institute for Life Sciences, Asan Medical Center, Seoul 05505, Republic of Korea; b University of Ulsan College of Medicine, Seoul 05505, Republic of Korea; c Department of Pathology, Asan Medical Center, Seoul 05505, Republic of Korea; d Graduate School of Medical Science and Engineering, KAIST, Daejeon 34141, Republic of Korea; e OncoClew Life Science Co., Ltd, Seoul 05505, Republic of Korea

Dysregulation of the Hippo pathway has been frequently identified in various human cancers. The Hippo pathway is a highly complex pathway involving multiple types of proteins, and the activation of YAP by LATS kinase is the final effector step in the transcription process. In this study, we linked the roles of the multifunctional adapter proteins β-arrestins (β-arrestin 1 and 2) in cooperation with other signaling pathways such as GPCR and Wnt to essential cellular functions involved in carcinogenesis, including the regulation of cell proliferation, migration, and differentiation as well as stem cell properties.^1^ Although β-arrestin 1 and 2 have high structural similarities, they have different roles in carcinogenesis as β-arrestin 1 aids cancer cell survival and metastasis^2^ and β-arrestin 2 inhibits tumor growth.^1^ In the Hippo signaling pathway, β-arrestins function as scaffold proteins that mediate the phosphorylation of key molecules, and their association with human cancers is a major research topic. In this study, we demonstrated that β-arrestin 2 inhibits YAP activation through the formation of the β-arrestin 2-LATS-YAP trimeric complex, which results in the promotion of the kinase activity of LATS in cancer cell lines and patient-derived colon cancer organoids.

We first examined the effect of β-arrestin 2 overexpression in A549 and RPE cells. Overexpression of β-arrestin 2 resulted in an increase in YAP phosphorylation (pYAP) (Fig. 1A; Fig. S1A, B), which in turn reduced the nuclear accumulation of YAP (Fig. S1B, C) and the expression of YAP transcriptional targets *ANKRD*, *CTGF* and *CYR61* was downregulated (Fig. S1D). Conversely, knockdown of endogenous β-arrestin 2 showed opposite results (Fig. 1B; Fig. S2A–C). As expected, YAP transcriptional activity was upregulated after the knockdown of β-arrestins (Fig. S2D). These effects were more pronounced in knockdown of β-arrestin 2 than β-arrestin 1 (Fig. 1B; Fig. S2). Next, to investigate the effect of β-arrestin 2 on YAP under the activation of Hippo signaling, we treated β-arrestin 2-knockdown cells with palmitic acid (PA) to stimulate metabolic signaling.^3^ The treatment of PA suppressed the nuclear translocation of YAP but β-arrestin 2-knockdown cells showed more nuclear YAP expression than controls (Fig. S3A). These data suggest that loss of β-arrestin 2 prevents YAP inactivation induced by metabolic signals.

**Figure 1:**
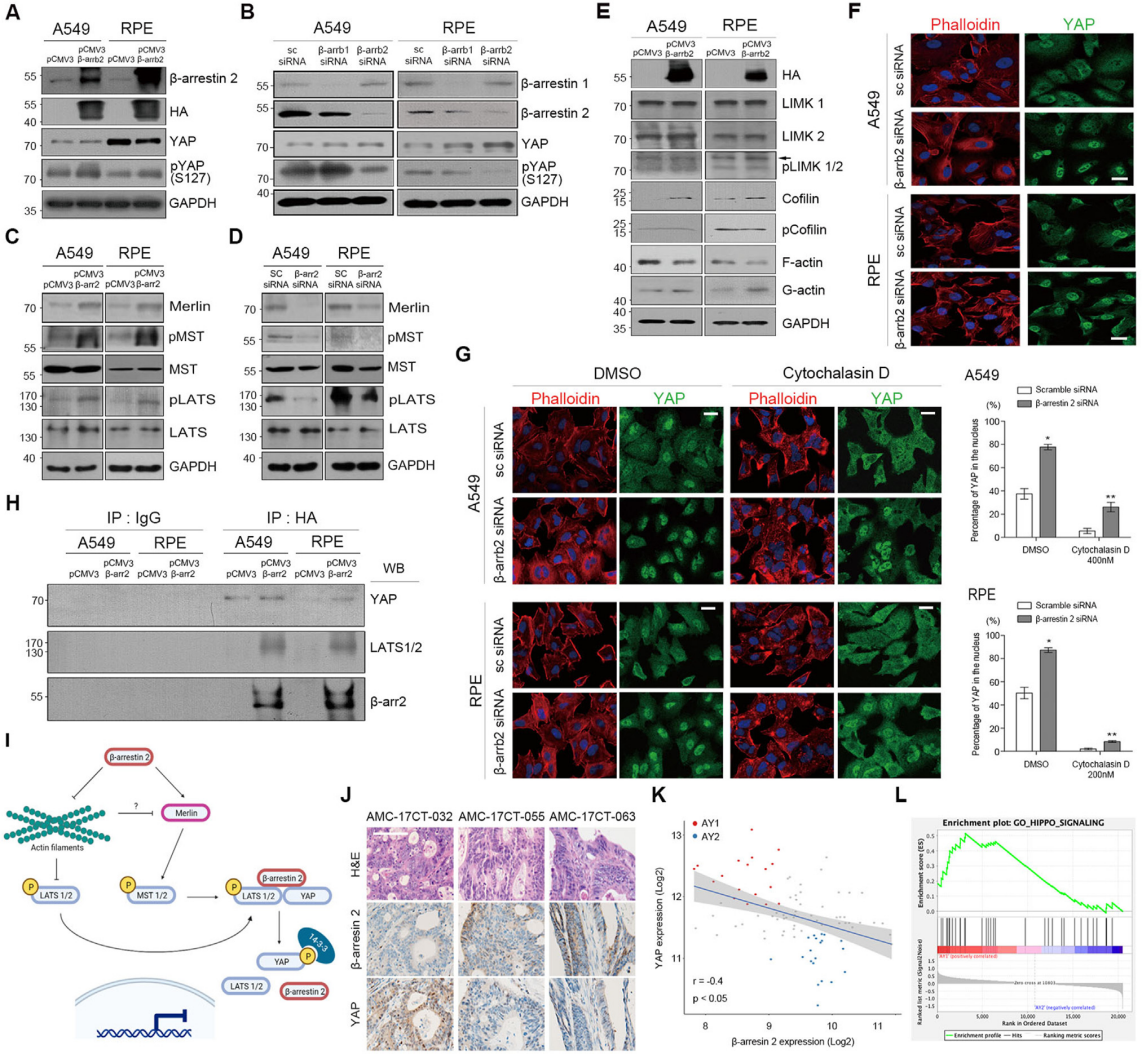
β-Arrestin 2 inhibits the activation of YAP through a dual mechanism. **(A)** Immunoblot analysis of β-arrestin 2 (β-arrb2), YAP and pYAP at Ser127 (S127) based on β-arrestin 2 overexpression (HA). GAPDH was used as a loading control. **(B)** Immunoblot analysis of β-arrestin 1 (β-arrb1) and 2 (β-arrb2), YAP and pYAP S127 after transfection with a small-interfering RNA (siRNA) specific for β-arrestin 1 or 2. GAPDH was used as a loading control (sc siRNA: scrambled siRNA). **(C, D)** Immunoblot analysis on the changes of several Hippo pathway cascade components after overexpression (C) and loss (D) of β-arrestin 2. GAPDH was used as a loading control. **(E)** Immunoblot analysis of the mechanism of actin remodeling regulated by β-arrestin 2. GAPDH was used as a loading control. **(F)** Immunofluorescence images showing the changes in YAP localization and actin remodeling based on the knockdown of β-arrestin 2 expression (scale bar: 20 μm). F-actin (red) was stained with phalloidin. Nuclei (blue) were stained with DAPI. **(G)** Immunofluorescence images showing the effect of an actin inhibiting drug (Cytochalasin D) on actin filament and YAP localization in cells transfected with β-arrestin 2 siRNA (scale bar: 20 μm) and their quantification graphs (right) (*n* = 3; error bars indicate SEM; *p*-value was calculated by paired *t*-test). Cells transfected with β-arrestin 2 siRNA or scramble siRNA (sc siRNA) were treated with dimethyl sulfoxide (DMSO) or cytochalasin D for 6 h. Detailed data of Fig. 1G are shown in Supporting Information (*, *P* < 0.05; **, *P* < 0.01; Table S2). **(H)** Immunoprecipitation analysis on the interaction of β-arrestin 2 with YAP and LATS after overexpression of β-arrestin 2 using anti-HA antibody. **(I)** A schematic illustration of the mechanism underlying the LATS kinase activation by β-arrestin 2. **(J)** Immunohistochemical analysis of β-arrestin 2 and YAP staining in colon tissues obtained from patients with colon cancer (scale bar: 100 μm). **(K)** Correlation of YAP and β-arrestin 2 RNA expression levels in 90 colon cancer organoids (CCOs). According to the RNA expression levels of β-arrestin 2 and YAP, CCOs with low expression of β-arrestin 2 and high expression of YAP were designated as the AY1 group (red dots, *n* = 18) and those with high expression of β-arrestin 2 and low expression of YAP were designated as the AY2 group (blue dots, *n* = 20). (Linear regression test; *r* = −0.4, *P* < 0.05) **(L)** Gene set enrichment analysis comparing the alterations in the Hippo pathway signaling between the AY1 and AY2 groups. Full-length blots are shown in Supporting Information (Fig. S6, S7), and the gels were run under the same experimental conditions.

Next, to investigate how β-arrestin 2 increases the kinase activity of LATS phosphorylating YAP, we screened the canonical Hippo signaling pathway. Overexpression of β-arrestin 2 resulted in an increase of Merlin (Fig. 1C), which directly activates MST.^4^ As expected, β-arrestin 2 overexpression increased the levels of pMST and pLATS (Fig. 1C). Conversely, β-arrestin 2-knockdown cells showed opposite results (Fig. 1D). These results suggest that β-arrestin 2 promotes the expression of Merlin to increase LATS activation.

In the GPCR signaling pathway, β-arrestins mediate actin remodeling, which in turn controls the kinase activity of LATS.^1^^,^^3^ When the amount of F-actin decreases, angiomotin (AMOT) released from F-actin promotes Merlin activation.^4^ Therefore, we investigated whether this negative role of β-arrestin 2 is associated with actin remodeling. β-Arrestin 2-positive cells displayed reduced F-actin through cofilin-mediated actin depolymerization independent of LIMK while showing an increase in G-actin levels (Fig. 1E). Immunofluorescence data also confirmed the lower levels of stress fiber formation in β-arrestin 2-positive cells (Fig. S4A). Conversely, the level of stress fiber formation was enhanced in β-arrestin 2-downregulated cells showing nuclear localization of YAP (Fig. 1F).

Disruption of stress fiber formation after treatment with cytochalasin D in β-arrestin 2-knockdown cells resulted in reduced nuclear YAP expression (Fig. 1G). Interestingly, some β-arrestin 2-knockdown cells still showed nuclear YAP expression despite the abortion of actin polymerization (Fig. 1G), suggestive of the existence of another mechanism underlying LATS kinase activation. Therefore, we focused on the scaffolding role of β-arrestin 2. Co-immunoprecipitation analyses using A549, RPE, and HEK293T cells free of mutations in the Hippo pathway genes showed that β-arrestin 2 could bind pLATS and YAP in these cells but fails to interact with pYAP (Fig. S4B, C). Next, we reconfirmed the direct interaction of β-arrestin 2 with LATS and YAP. In immunofluorescence analysis, β-arrestin 2 was co-localized with YAP and LATS (Fig. S4D, E); moreover, co-immunoprecipitation of exogenous β-arrestin 2 directly showed that β-arrestin 2 binds LATS and YAP (Fig. 1H). However, binding was weakened after the loss of β-arrestin 2 (Fig. S4F). Importantly, YAP phosphorylation mediated by β-arrestin 2 was closely associated with Ser127(S127) rather than Ser397(S397) in YAP (Fig. S5A, B). Therefore, the binding of YAP to 14-3-3 increased after overexpression of β-arrestin 2 (Fig. S5C) but without inducing significant changes in the level of 14-3-3 (Fig. S5A, B). Additionally, to identify the binding regions between β-arrestin 2 and LATS/YAP, YAP with a deletion in the WW domain, which spans the S127 site, was expressed in A549 and RPE cells. Deletion of the WW domain resulted in decreased YAP and LATS binding (Fig. S5E) and decreased YAP phosphorylation without changes in YAP expression (Fig. S5D). However, the binding of β-arrestin 2 to YAP was not reduced in cells expressing the WW domain deletion form (Fig. S5E). These results collectively show that β-arrestin 2 forms a trimeric complex with LATS 1/2 and YAP and that this complex induces phosphorylation at S127 of YAP to increase the binding to 14-3-3. This novel mechanism of β-arrestin 2-mediated phosphorylation of YAP is independent of actin remodeling.

Our aforementioned experiments revealed three different mechanisms for LATS kinase activation by β-arrestin 2 (Fig. 1I). First, β-arrestin 2 promotes the activation of Merlin to directly activate MST and LATS. Second, β-arrestin 2 inhibits actin polymerization, which leads to LATS activation through dephosphorylation of cofilin, a mediator of actin cytoskeletal dynamics that binds to flanking F-actin filaments and contributes to F-actin depolymerization. This mechanism also affects the activation of Merlin. Third, β-arrestin 2 serves as a direct scaffold to form a trimeric complex with LATS and YAP to further promote YAP phosphorylation by activated LATS. Under these conditions, LATS phosphorylates YAP at S127, which is recognized by the 14-3-3 protein, induces cytoplasmic retention of YAP, and consequently inhibits its activation.

As YAP activation has been implicated in a variety of human cancers, β-arrestin 2 may possibly act as a tumor suppressor. We demonstrated the role of β-arrestin 2 in the inhibition of YAP activation in colon cancer tissues and CCO lines (Table S1), which recapitulate the characters of human colon cancer tissues.^5^ Immunohistochemistry revealed high nuclear YAP levels in tissues with low expression of β-arrestin 2; in contrast, those with high β-arrestin 2 had low nuclear YAP expression (Fig. 1J). In the RNA-sequencing data, YAP and β-arrestin 2 expression levels showed a reverse correlation (Fig. 1K). Based on the level of YAP expression, 38 CCOs showing strong reverse correlations were subgrouped into AY1 (low β-arrestin 2 + high YAP) and AY2 (high β-arrestin 2 + low YAP) (Fig. 1K). Gene set enrichment analysis showed that the Hippo pathway signaling was lower in the AY2 than in the AY1 (Fig. 1L). These observations support the idea that β-arrestin 2 negatively regulates YAP expression and its activation in colon cancer tissues and organoids.

In conclusion, our study reveals a novel function of β-arrestin 2 forming a trimeric complex with LATS and YAP through its scaffold function, resulting in LATS-mediated phosphorylation of YAP. Our results provide a new insight for targeting the Hippo pathway in human cancers.

## Author contributions

SJJ, MK, and JK conceived and designed the study; MK and JMK performed the experiments; COS and EJC analyzed and studied the whole genomic data; SJJ, MK, JMK, EJC, COS, and JK analyzed the data; and SJJ, MK, and COS wrote the manuscript with contributions from all co-authors. All authors read and approved the final manuscript.

## Conflict of interests

The authors declare that they have no conflict of interest.

## Funding

This study was supported by the Technology Innovation Program for Fostering New Post-Genome Industry funded by the Ministry of Trade, Industry & Energy (MOTIE, Korea) (No. 10067796 and 10067407) and by the National Research Foundation of Korea (NRF) grant funded by the Ministry of Science and ICT (MSIT, Korea) (No. NRF-2020R1C1C1004935).

## References

[1] Bagnato A., Rosanò L. (2019). New routes in GPCR/β-arrestin-driven signaling in cancer progression and metastasis. Front Pharmacol.

[2] Tocci P., Cianfrocca R., di Castro V. (2019). β-arrestin1/YAP/mutant p53 complexes orchestrate the endothelin A receptor signaling in high-grade serous ovarian cancer. Nat Commun.

[3] Yamaguchi H., Taouk G.M. (2020). A potential role of YAP/TAZ in the interplay between metastasis and metabolic alterations. Front Oncol.

[4] Li Y., Zhou H., Li F. (2015). Angiomotin binding-induced activation of Merlin/NF2 in the Hippo pathway. Cell Res.

[5] Cho E.J., Kim M., Jo D. (2021). Immuno-genomic classification of colorectal cancer organoids reveals cancer cells with intrinsic immunogenic properties associated with patient survival. J Exp Clin Cancer Res.

