# Supplementary material for: β-Arrestin 2 suppresses the activation of YAP by promoting LATS kinase activity
Source: Genes Dis. 2022 May 17;10(2):348–51. doi: 10.1016/j.gendis.2022.04.017 (PMC10201590; doi:10.1016/j.gendis.2022.04.017)
Supplement: Multimedia component 1 [file mmc1.docx]

Supplementary data for

**β-Arrestin 2 suppresses the activation of YAP by promoting LATS kinase activity**

Minsuh Kim ^a^, Ji Min Kim ^b^, Eun Jeong Cho ^b^, Chang Ohk Sung ^b,c^, Joon Kim ^d^, Se Jin Jang ^b,c,e,*^

^a^ Asan Institute for Life Sciences, Asan Medical Center, Seoul 05505, Republic of Korea

^b^ University of Ulsan College of Medicine, Seoul 05505, Republic of Korea

^c^ Department of Pathology, Asan Medical Center, Seoul 05505, Republic of Korea

^d^ Graduate School of Medical Science and Engineering, KAIST, Daejeon 34141, Republic of Korea

^e^ OncoClew Life Science Co., Ltd, Seoul 05505, Republic of Korea

***Corresponding author**:

Se Jin Jang, MD, PhD

Department of Pathology

Asan Medical Center, University of Ulsan College of Medicine

88, Olympic-ro 43-gil, Songpa-gu, Seoul 05505, Republic of Korea

Tel: +82-2-3010-5966

E-mail: [jangsejin@amc.seoul.kr](mailto:jangsejin@amc.seoul.kr)

**This file includes:**

Methods and Materials

Fig. S1-7

**Materials and Methods**

**Cell culture and drug treatment**

RPE cells were cultured in Dulbecco’s modified Eagle’s medium (DMEM)/F12 (Lonza, Basel, Switzerland), and A549 cells were cultured in Roswell Park Memorial Institute (RPMI)-1640 medium (Gibco, CA, USA). HEK293T cells were cultured in DMEM (Gibco). All culture media were supplemented with 10% fetal bovine serum (FBS; Gibco) and 1% penicillin/streptomycin (Gibco). Palmitic acid (PA; Sigma, MO, USA), the most common saturated fatty acid, was used to stimulate metabolism signals. PA preparation and treatment were performed as described previously.^1^ Cytochalasin D (Sigma, MO, USA) was used to disrupt the formation of actin filaments.

**Transfection, small-interfering RNAs (siRNAs), and DNA construct**

Plasmid transfection was performed using Lipofectamine 2000 (Invitrogen, MA, USA) according to the manufacturer’s protocol. Plasmids expressing β-arrestin 2 (pCMV3 β-arrestin 2 HA) or negative control (pCMV3 HA) were generated by Sino Biological (Beijing, China) and plasmid for identifying the binding regions between β-arrestin 2 and LATS/YAP (YAP WT; pMSV-puroflag-YAP 5SA, YAP ΔWW; pMSV-puroflag-YAP 5SA-dww) were provided by Dr. Dae-Sik Lim (Korea Advanced Institute of Science and Technology). The overexpression efficiency was determined by western blotting at 24 h or 48 h after transfection.

siRNA transfection was performed using Lipofectamine RNAiMax (Invitrogen) at a final concentration of 20 nM according to the manufacturer’s protocol. The following duplex siRNAs were used: β-arrestin 1, 5′-AAA GCC UUC UGC GCG GAG A-3′; β-arrestin 2, 5′-AAG GAC CGC AAA GUG UUU G-3′. The knockdown efficiency was determined by western blotting and reverse-transcriptase quantitative polymerase chain reaction (RT-qPCR) at 48 h after transfection.

**Immunofluorescence**

Cells were fixed in 4 % paraformaldehyde for 15 min, permeated with 0.5 % Triton X-100/phosphate-buffered saline (PBS) for 5 min, and blocked with 3 % bovine serum albumin/PBS. Then, the cells were incubated overnight at 4 °C with primary antibodies against YAP (1:1000; Santa Cruz Biotechnology, TX, USA) or HA (1:200; Abcam, MA, USA), followed by incubation with Alexa Fluor 488- and 594-conjugated secondary antibodies (1:1000; Invitrogen) for 1 h at room temperature. For visualization of F-actin, the cells were labeled with Alexa-phalloidin (1:1000; Invitrogen) for 1 h at room temperature. Nuclei were counterstained with 4′,6-diamidino-2-phenylindole (DAPI; 1:2000; Sigma) for 15 min, and fluorescence signals were visualized using a Carl Zeiss fluorescence microscope (Carl Zeiss, Jena, Germany).

**Western blot analysis**

The cultured cells were harvested and lysed in a lysis buffer (Cell Signaling Technology, MA, USA) containing phosphatase inhibitor cocktail C (Santa Cruz Biotechnology). To fractionate the nuclear proteins, cells were lysed using the NE-PER Nuclear Cytoplasmic Extraction Reagent kit (Pierce Biotechnology, IL, USA). The concentration of proteins in cell lysates was determined using the Enhanced BCA Protein Assay Kit (Pierce Biotechnology). Generally, 20 μg of proteins were loaded in each lane of a gel; however, 50 μg of proteins were loaded for β-arrestin detection. The blots were incubated overnight at 4°C with the following primary antibodies: anti-β-arrestin 1/2 (1:1000; BioLegend Inc., CA, USA), anti-β-arrestin 1 (1:500; Abcam), anti-β-arrestin 2 (1:1000; Cell Signaling Technology), anti-large tumor suppressor (LATS) 1/2 (1:1000; Abcam), anti-phosphorylated LATS (pLATS1; 1:1000; Cell Signaling Technology), anti-YAP/TAZ (1:1000; Santa Cruz Biotechnology), anti-phosphorylated YAP (pYAP S127 and S397; 1:1000; Cell Signaling Technology), anti-HA (1:1000; Abcam), anti-histone deacetylase (HDAC, 1:1000; Cell Signaling Technology), anti-LIMK 1 and 2 (1:500; Santa Cruz Biotechnology), anti-phosphorylated LIMK 1/2 (pLIMK1/2; 1:500; Cell Signaling Technology), anti-cofilin (1:1000; Santa Cruz Biotechnology), anti-phosphorylated cofilin (pcofilin; 1:1000; Cell Signaling Technology), anti-F- and G-actin (1:1000; Abcam), anti-14-3-3 (1:1000; Cell Signaling Technology), and anti-glyceraldehyde 3-phosphate dehydrogenase (GAPDH, 1:5000; Santa Cruz Biotechnology). Incubation with horseradish peroxidase (HRP)-conjugated goat anti-rabbit or anti-mouse IgG secondary antibodies (1:1000; Enzo Life Sciences, Inc., NY, USA) was performed for 1 h at room temperature. The full blots of the cropped images are provided in Fig. S6, S7.

**RT-qPCR**

Trizol (Invitrogen) was used for the extraction of total RNA, and all RNA samples were treated with DNase (Promega, WI, USA). cDNA was synthesized using a High Capacity cDNA Reverse Transcription Kit (Applied Biosystems, CA, USA). The expression levels of target genes were quantified using target-specific primers. The sequence information used for RT-qPCR is provided in Table S6. RT-PCR was performed using the CFX Connect Real-Time PCR Detection System (Bio-Rad, CA, USA).

**Immunoprecipitation**

For immunoprecipitation, cells were lysed in a lysis buffer (Cell Signaling Technology) containing phosphatase inhibitor cocktail C (Santa Cruz). The Enhanced BCA Protein Assay Kit (Pierce Biotechnology) was used to quantify the concentration of proteins in cell lysates, and 1 mg of proteins were used for immunoprecipitation. Then, 5 μL antibody against β-arrestin 1/2 (BioLegend), 3 μL antibody specific for LATS 1/2 (Abcam), 10 μL antibody for YAP/TAZ (Santa Cruz), and 5 μL antibody against immunoglobulin G (IgG) (Santa Cruz; control group) per mg/mL lysate were incubated overnight at 4 °C with constant rotation. The next day, protein G Sepharose beads (GE Healthcare Bio-Sciences Corp, Buckinghamshire, UK) were added to each lysate for 4 h at 4 °C with constant rotation. Unbound proteins were removed by washing four times using the lysis buffer, and the bound proteins were eluted from the beads by adding 40 µL of a sample buffer. Sodium dodecyl sulfate polyacrylamide gel electrophoresis (SDS-PAGE) was performed using 20 µL samples.

**Immunohistochemistry**

Tissues and organoids were fixed in 4% paraformaldehyde and then subjected to dehydration, paraffin embedding, sectioning, and standard hematoxylin and eosin (H&E) staining. For immunohistochemical staining, samples were incubated with the following primary antibodies: anti-β-catenin 2 (1:100; Cell Signalling Technology), anti-YAP (1:500; Santa Cruz), anti-keratin 20 (CK20; 1:400; Dako, CA, USA), and anti-CDX2 (1:1000; Cell Signalling Technology). The sections were subsequently incubated with the corresponding secondary antibodies (1:5000; Vector Laboratories, CA, USA) and visualized using the ultraView Universal DAB Detection kit (Ventana Medical Systems). Nuclei were counterstained with Harris hematoxylin. Images were acquired using the CELENA X System (Logos Biosystems, Anyang, Republic of Korea).

**Human tissue preparation and culture of colon cancer organoids (CCOs)**

Small pieces (approximately 1–4 cm^3^) of colon cancer tissues were obtained from surgically resected colon specimens as part of the colon cancer biobanking process at the Asan Bio-Resource Center (Seoul, Republic of Korea) after obtaining informed consent from the patients. The samples were placed in cold Hank’s balanced salt solution (HBSS, Lonza) with 1× Primocin (InvivoGen, Hong Kong, China) and transported to the laboratory on ice within 1 h of excision from patients. Samples were washed thrice with cold HBSS with antibiotics and sectioned into approximately 1–2 mm^3^ sized segments using sterile blades. Five-to-six pieces of each sectioned sample were incubated with 0.2 U/μL collagenase II (Gibco), 1% penicillin/streptomycin (Gibco), and 0.5 mg/mL amphotericin B (2 % antibiotics, Sigma) in DMEM/F12 medium (Gibco) at 37 °C for 40–90 min with intermittent agitation. After incubation, the suspensions were repeatedly triturated by pipetting, centrifuged at 161 rcf for 5 min, and washed thrice with DPBS (Welgene, Seoul, Republic of Korea). Next, the suspensions were passed through 100 μm cell strainers (BD Falcon, CA, USA) and centrifuged at 40 rcf for 3 min, and the resulting pellets were resuspended in 100 μL minimum basal medium for colorectal cancer organoid (CCO MBM), which is a serum-free medium (DMEM/F12; Gibco) supplemented with 50 ng/mL human epidermal growth factor (Invitrogen), B27 (Invitrogen), 1 mM n-acetylcysteine (PeproTech, NJ, USA), 10 mM nicotinamide (PeproTech), 10 nM gastrin I (PeproTech), 500 nM A83-01 (PeproTech), 10 μM ROCK inhibitor (PeproTech), and 1 % penicillin/streptomycin (Gibco).

To establish organoids, 200 μL Matrigel (Corning, NY, USA) was added to the remaining 100 μL suspension, and 150 μL of the resulting cell suspension was allowed to solidify for 10 min at 37 °C in a well of a pre-warmed six-well culture plate (Corning). After gelation, 3 mL CCO MBM was added to the well. The medium was changed every 3 - 4 days, and the organoids were passaged after 1–3 weeks. For passaging, a solidified Matrigel drop containing the organoids was harvested using cold DPBS and centrifuged at 112 rcf for 3 min at 4 °C. The pellets were washed with cold DPBS and centrifuged at 250 rcf for 15 min at 4 °C. The organoids were resuspended in 2 mL TrypLE Express (Invitrogen) and incubated for 10 min at 37 °C for dissociation. Later, 10 mL of DMEM/F12 containing 10 % FBS was added, and the samples were centrifuged at 112 rcf for 3 min. The pellets were washed with DPBS and centrifuged at 112 rcf for 3 min. The pellets were resuspended in CCO MBM + Matrigel (1:3) and reseeded at 1:3 to 1:4 ratios to allow the formation of new CCOs.

The research protocol was approved by the Institutional Review Board of the Asan Medical Center (#2018-0152; Seoul, Republic of Korea). The entire experimental protocol was conducted in compliance with the institutional guidelines. All samples were confirmed as cancers on the basis of histopathological assessment. The diagnosis of each case was confirmed by pathologists at Asan Medical Center.

**Whole-transcriptome sequencing and data processing**

Total RNA sequencing was performed on 90 CCOs and 88 matched formalin-fixed and paraffin-embedded (FFPE) primary tumor tissues. For FFPE tissues, manual microdissection of unstained tissue sections under a light microscope was performed for viable tumor areas without normal proper muscle using corresponding H&E slides as a reference. Total RNA was extracted using the RNeasy Mini Kit (Qiagen, Hilden, Germany) according to the manufacturer’s protocol. A cDNA library was constructed using the TruSeq RNA Access Library Prep Kit (Illumina, Inc., CA, USA) and 1 mg of total RNA. All cases passed the cDNA library quality assurance (minimum requirement: > 5 nM). Finally, 100-nt paired-end sequencing was performed using the HiSeq 2500 platform (Illumina, Inc.). The RNA sequencing data for primary cancer tissues and CCOs were processed in the same manner as follows: raw RNA-seq data were analyzed using the TCGA RNA-seq Pipeline (v2) after sequencing quality assurance, and the quality of the FastQ files was checked using FastQC (https://github.com/s-andrews/FastQC, v0.7.15). The fastQC’s “basic statistics” results of all primary cancer tissues and organoids were “PASS.” Primary cancer tissues had the following average basic statistics: total sequence, 50391531.95; GC, 47.97 %; total deduplicated percentage, 23.98 %. The CCOs had the following average basic statistics: total sequence 43461472.52; GC, 50.18 %; total deduplicated percentage, 19.85 %. The TCGA RNA-seq Pipeline (v2) was used to analyze the raw RNA-seq reads and quantify the gene expression. Reads that passed the quality check were mapped to the human reference genome (hg19) using MapSplice v2.2.1 ^2^. RSEM v1.3.0 ^3^ was used for transcript quantification and normalized within samples to a fixed upper quartile.

**Pathway analysis**

Gene set enrichment analysis (GSEA v4.0.2) ^4^ was used to identify alterations in the Hippo signaling pathway among various groups in the RNA expression profile of the CCOs. The gene set used was GO_HIPPO_SIGNALING, and GSEA results were considered significant when the FDR *q* value was < 0.05 and the nominal *p* value was < 0.05.

**Statistical analysis**

Data are expressed as mean ± standard error of the mean from at least three independent experiments. Statistical analysis was performed using SPSS Version 24 and R version 4.0.2 (R Foundation for Statistical Computing, Vienna, Austria). The graphs were generated using GraphPad Prism 5. *p* values < 0.05 were considered to indicate statistically significant differences.

**Supplementary Figures**

**
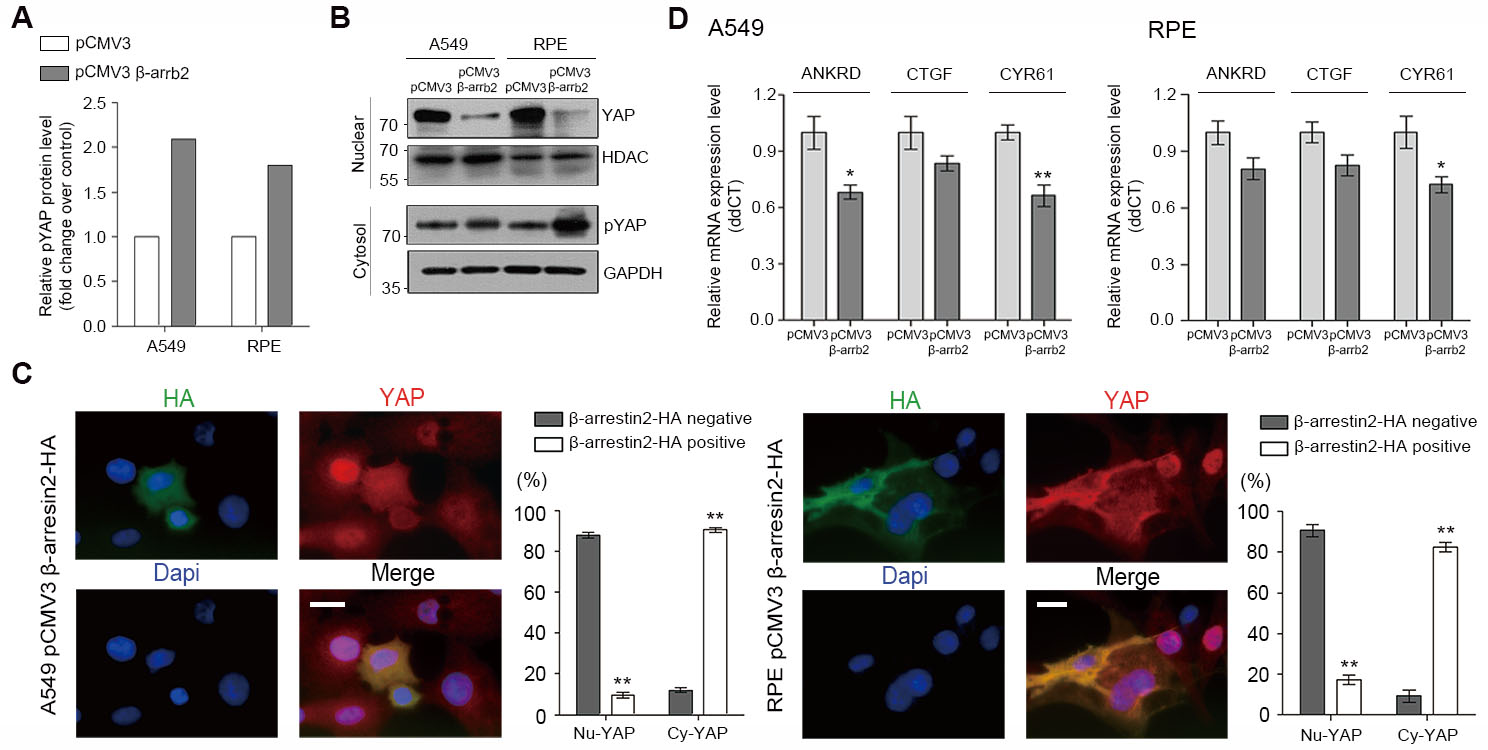
**

**Figure S1.** β-Arrestin 2 inhibits the transcriptional activity and nuclear translocation of YAP. **(A)** Quantification graphs of pYAP protein levels according to β-arrestin 2 overexpression (pCMV-β-arrb2). The protein levels were quantified by western blotting in Fig. 1A using whole lysates and measured as fold change over control after being normalized to GAPDH level using ImageJ. **(B)** Nuclear and cytoplasmic fractionation of A549 and RPE cells overexpressing β-arrestin 2. The relative levels of YAP in the nucleus and pYAP in the cytoplasm were normalized using HDAC for proteins in the nucleus or GAPDH for those in the cytoplasm. **(C)** Immunofluorescence images of YAP in cells overexpressing β-arrestin 2 (β-arrestin2-HA positive, scale bar: 20 μm) and their quantification graphs (right) (*n* = 3; Nu-YAP: nuclear YAP; Cy-YAP: cytoplasmic YAP; error bars are standard errors of the mean [SEM]; *p*-value was calculated by paired *t*-test). Nuclei (blue) were stained with DAPI. Detailed data are provided in Table S3. **(D)** RT-qPCR analysis of the transcriptional targets of YAP according to the overexpression of β-arrestin 2. β-Actin was used as an internal control for RT-qPCR (*n* = 3; error bars indicate SEM; *p*-value was calculated by two-way ANOVA). *, *p* < 0.05; **, *p* < 0.01; *n.s.* non-significant, pCMV3 *vs* pCMV3 β-arrb2.

Full-length blots are shown in Fig. S7, and the gels were run under the same experimental conditions.


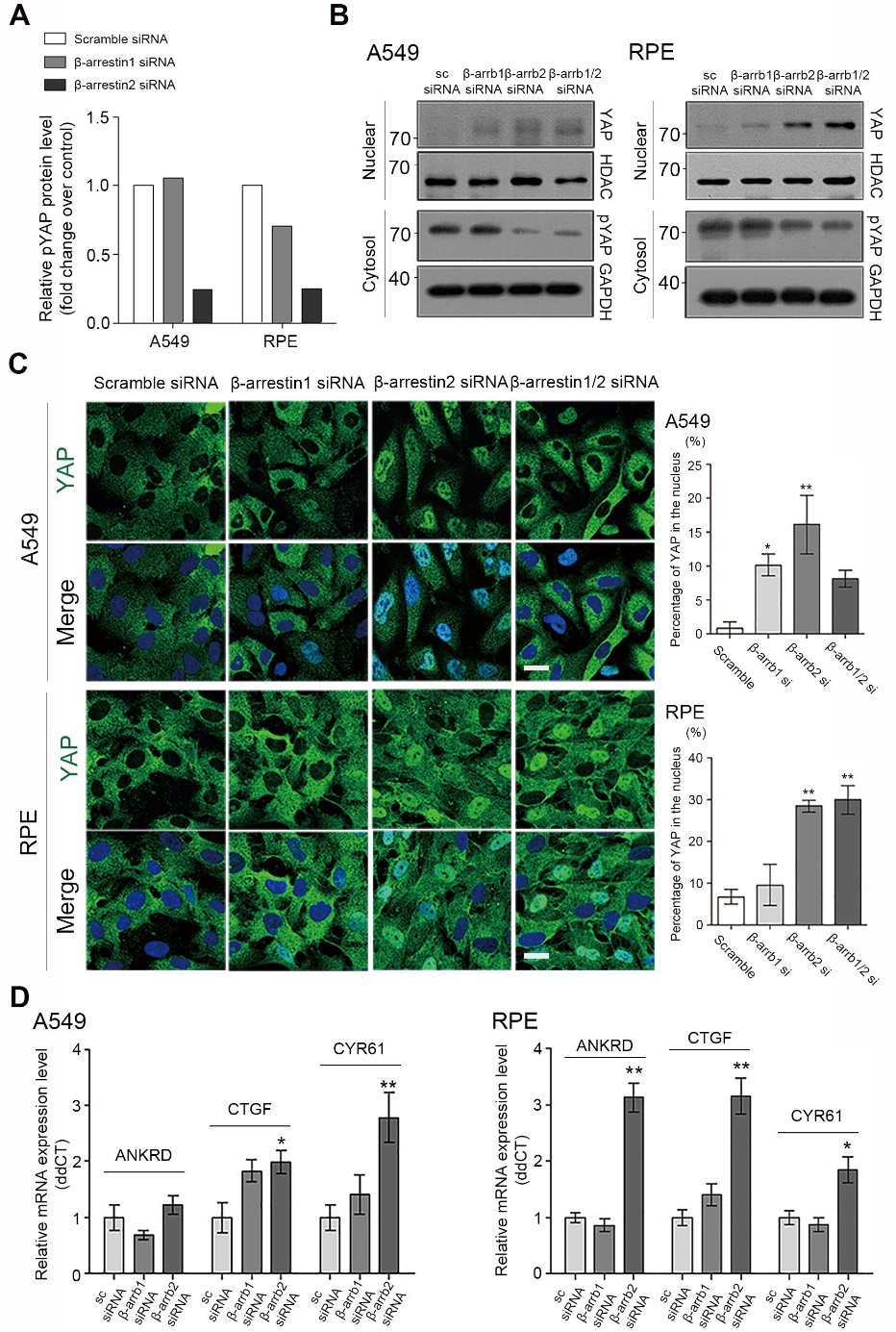


**Figure S2.** β-Arrestin knockdown promotes the activation of YAP. **(A)** Quantification of pYAP protein levels according to the knockdown of β-arrestin 1 or 2. The protein levels in whole lysates were quantified with Western blotting (Fig. 1B) and measured as fold change over control after being normalized to GAPDH using ImageJ. **(B)** Nuclear and cytoplasmic fractionation of A549 and RPE cells transfected with siRNAs against β-arrestin 1 and/or 2. The relative levels of YAP in the nucleus and pYAP in the cytoplasm were normalized to HDAC for proteins in the nucleus or GAPDH for proteins found in the cytoplasm. **(C)** Immunofluorescence images of cells transfected with siRNAs against β-arrestin 1 (β-arr 1) and/or 2 (β-arr 2) (scale bar: 20 μm) and their quantification graphs (right) (*n* = 3; error bars indicate SEM; *p*-value was calculated by paired *t*-test). Nuclei (blue) were stained with DAPI. Detailed data are provided in Table S4. *, *p* < 0.05; **, *p* < 0.01, Scramble (scrambled siRNA) vs β-arr 1 si (β-arrestin 1 siRNA), Scramble vs β-arrb2 si (β-arrestin 2 siRNA), Scramble vs β-arrb1/2 si (β-arrestin 1/2 siRNA). **(D)** RT-qPCR analysis of the transcriptional targets of YAP depending on the knockdown of β-arrestin 1 or 2. β-Actin was used as an internal control for RT-qPCR (*n* = 3; error bars indicate SEM; p-value was calculated by two-way ANOVA). *, *p* < 0.05; **, *p* < 0.01, sc siRNA *vs* β-arrb1 siRNA, sc siRNA *vs* β-arrb2 siRNA.

Full-length blots are shown in Fig. S7, and the gels were run under the same experimental conditions.


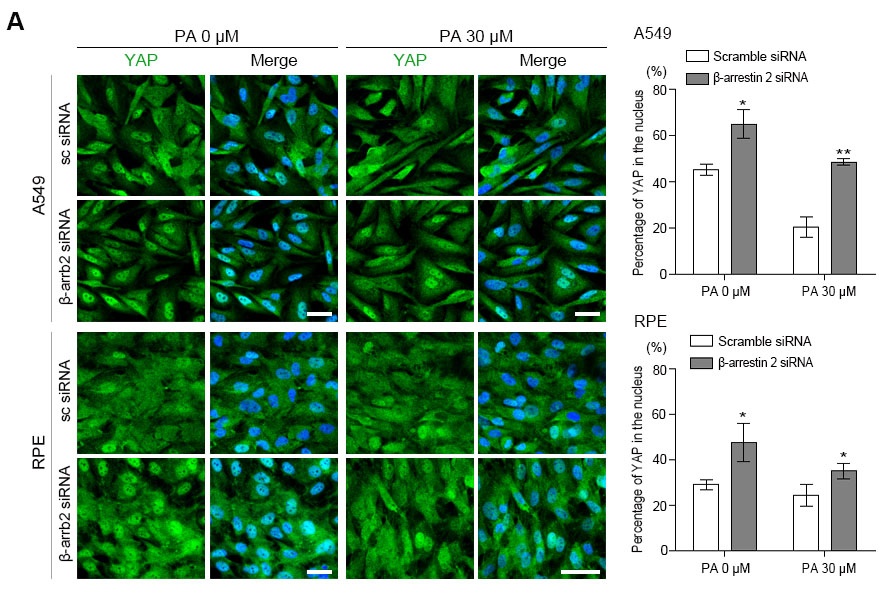


**Figure S3.** The knockdown of β-Arrestin 2 interrupts the blocking of YAP nuclear translocation by the treatment of PA **(A)** Immunofluorescence images of YAP localization after treating PA in cells transfected with sc siRNA (scrambled siRNA) and β-arrb 2 siRNA (β-arrestin 2 siRNA) (scale bar: 50 μm) and their quantification graphs (right) (*n* = 3; error bars indicate SEM; *p*-value was calculated by paired *t*-test). Nuclei (blue) were stained with DAPI. Detailed data are provided in Table S5. *, *p* < 0.05; **, *p* < 0.01.


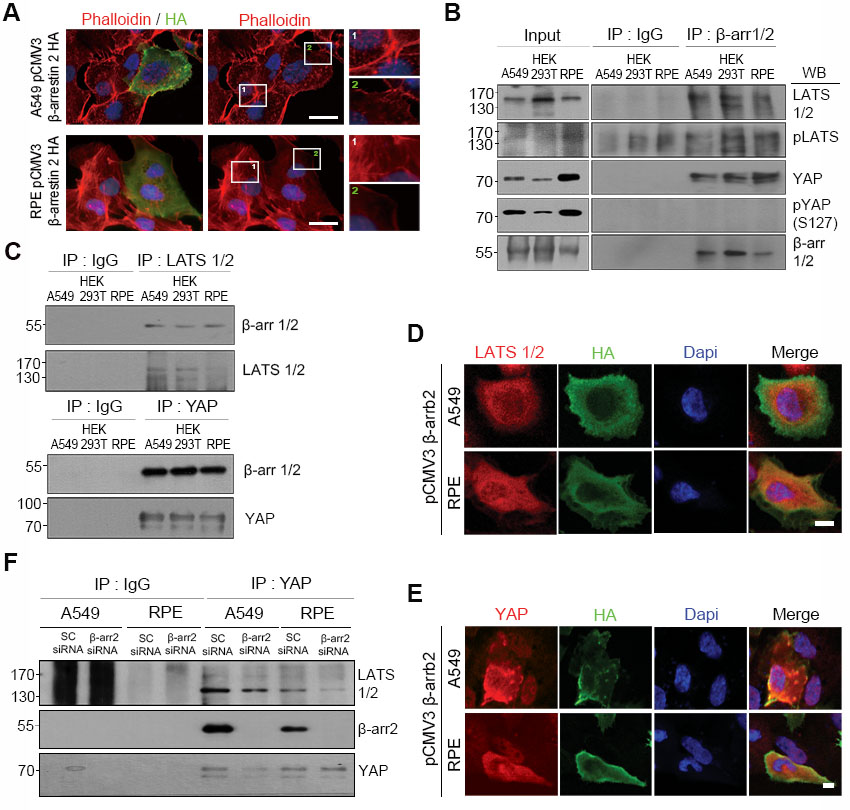


**Figure S4.** β-arrestin 2 induces the depolymerization of actin filaments and forms a complex with LATS and YAP to phosphorylate YAP **(A)** Immunofluorescence images of actin remodeling by β-arrestin 2 (β-arrb2) overexpression (scale bar: 10 μm). F-actin (red) was stained with phalloidin. HA staining (green) indicates β-arrestin 2 overexpression. Nuclei (blue) were stained with DAPI. **(B, C)** Immunoprecipitation analysis of β-arrestin 2, LATS, and YAP in A549, HEK293T, and RPE cells using antibodies against β-arrestin 2 **(B)**, LATS and YAP **(C)**. **(D, E)** Immunofluorescence images showing the co-localization of β-arrestin 2 with LATS **(D)** and YAP **(E)**. HA staining (green) indicates β-arrestin 2 overexpression. Nuclei (blue) were stained with DAPI (scale bar: 10 μm). **(F)** Immunoprecipitation analysis of β-arrestin 2, LATS, and YAP in cells transfected with sc siRNA (scrambled siRNA) and β-arrb 2 siRNA (β-arrestin 2 siRNA) using the antibody against YAP.

Full-length blots are shown in Fig. S7 and the gels were run under the same experimental conditions.

**
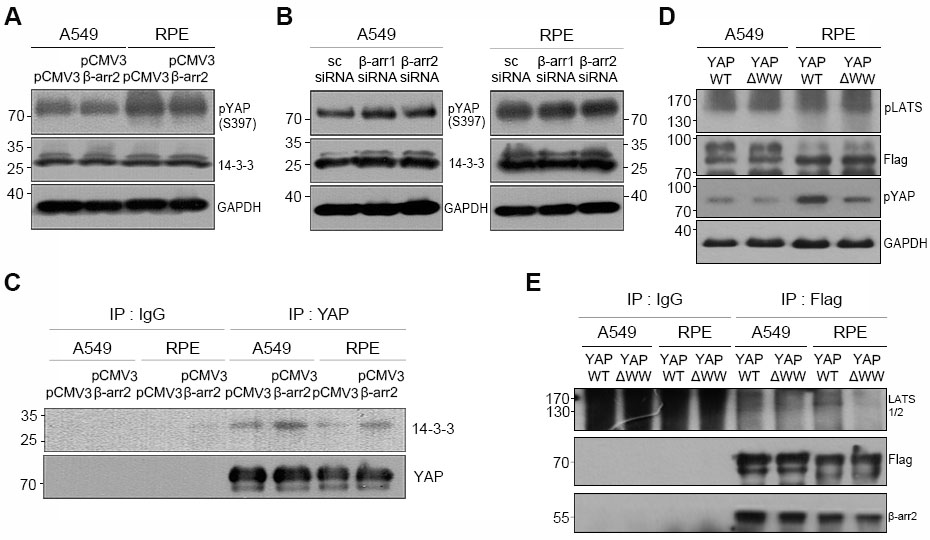
**

**Figure S5.** β-arrestin 2 induces phosphorylation at S127 of YAP **(A, B)** Immunoblot analysis of pYAP(S397) and 14-3-3 in cells with overexpression **(A)** or knockdown **(B)** of β-arrestin 2. GAPDH was used as a loading control. **(C)** Immunoprecipitation analysis of 14-3-3 and YAP in cells overexpressing β-arrestin 2. **(D)** Immunoblot analysis of the change of pYAP expression in cells transfected with vector deleted WW domain of YAP (YAP ΔWW; pMSV-puroflag-YAP 5SA-dww). Control cells were transfected with a vector expressing YAP tagged with flag (YAP WT; pMSV-puroflag-YAP 5SA). GAPDH was used as a loading control. **(E)** Immunoprecipitation analysis of β-arrestin 2, LATS, and YAP in cells transfected with YAP WT and ΔWW using the antibody against flag.

Full-length blots are shown in Fig. S7, and the gels were run under the same experimental conditions.


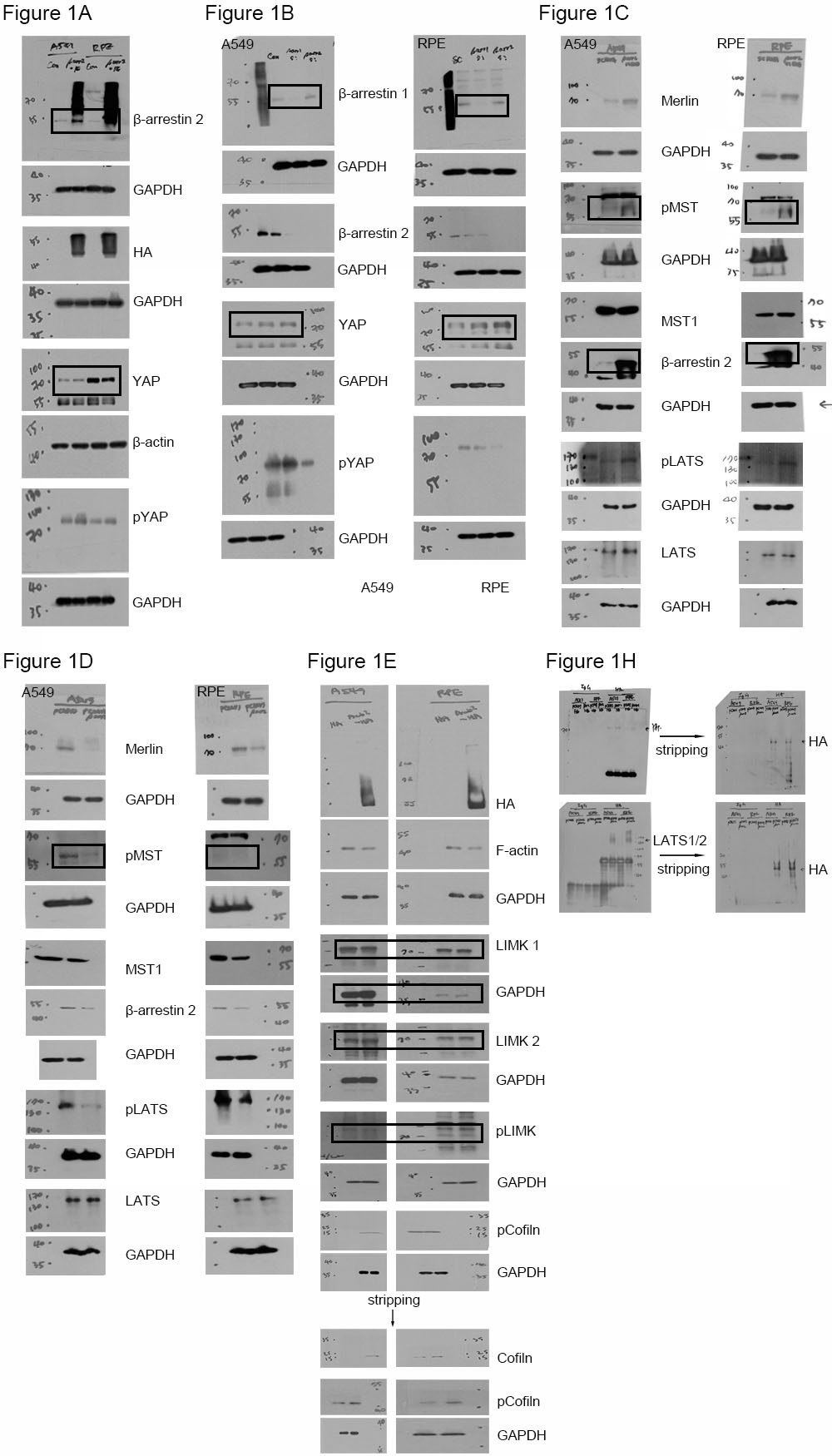


**Figure S6**


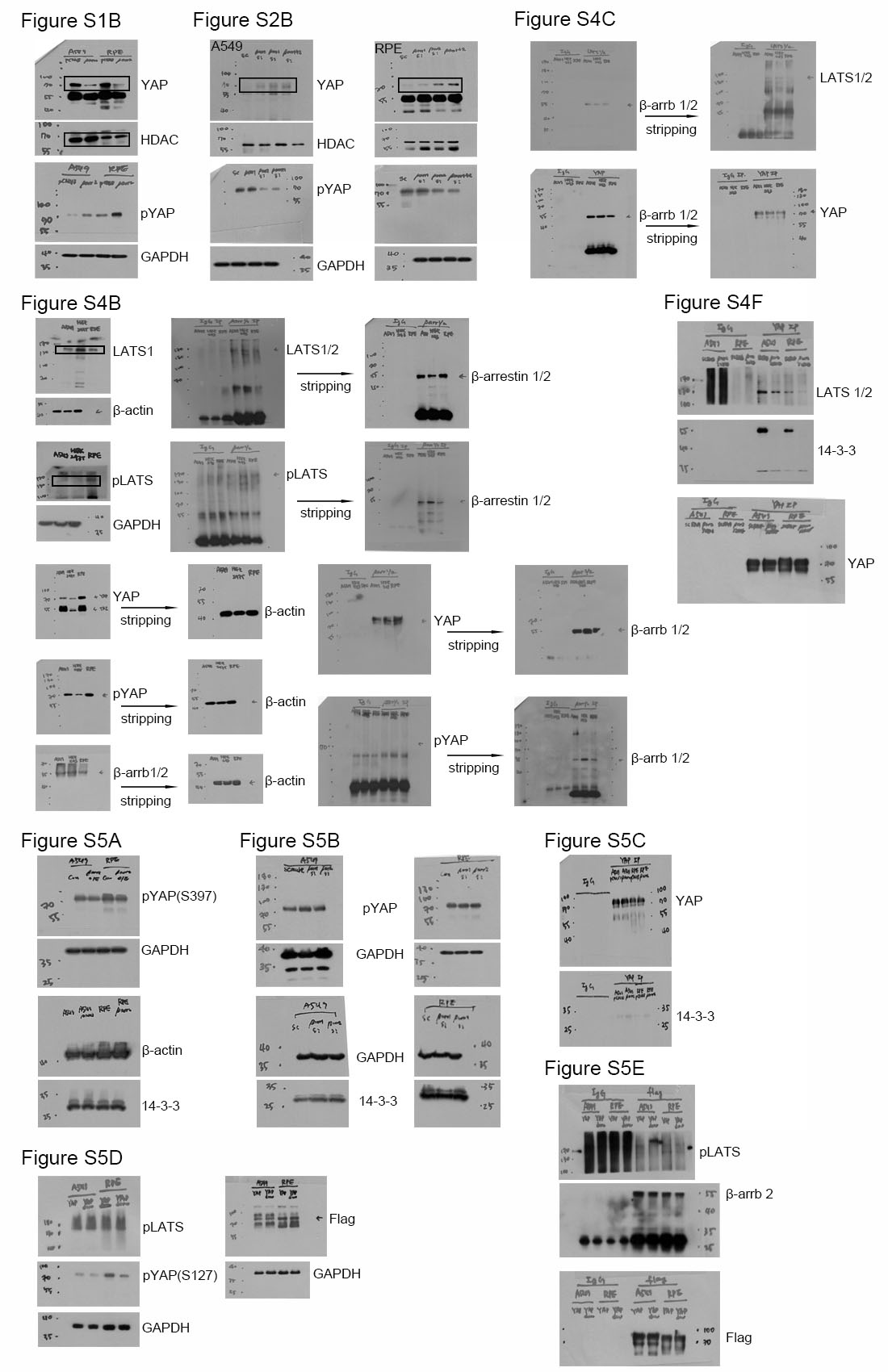


**Figure S7**

**Reference**

1. Yuan L, Mao Y, Luo W, et al. Palmitic acid dysregulates the Hippo-YAP pathway and inhibits angiogenesis by inducing mitochondrial damage and activating the cytosolic DNA sensor cGAS-STING-IRF3 signaling mechanism. *The Journal of biological chemistry*. Sep 8 2017;292(36):15002-15015.

2. Wang K, Singh D, Zeng Z, et al. MapSplice: accurate mapping of RNA-seq reads for splice junction discovery. *Nucleic Acids Res*. Oct 2010;38(18):e178.

3. Li B, Dewey CN. RSEM: accurate transcript quantification from RNA-Seq data with or without a reference genome. *BMC Bioinformatics*. Aug 4 2011;12:323.

4. Subramanian A, Tamayo P, Mootha VK, et al. Gene set enrichment analysis: a knowledge-based approach for interpreting genome-wide expression profiles. *Proc Natl Acad Sci U S A*. Oct 25 2005;102(43):15545-50.
